# Optimizing neuronal differentiation from induced pluripotent stem cells to model ASD

**DOI:** 10.3389/fncel.2014.00109

**Published:** 2014-04-11

**Authors:** Dae-Sung Kim, P. Joel Ross, Kirill Zaslavsky, James Ellis

**Affiliations:** ^1^Program in Developmental and Stem Cell Biology, The Hospital for Sick ChildrenToronto, ON, Canada; ^2^Department of Molecular Genetics, University of TorontoToronto, ON, Canada

**Keywords:** human pluripotent stem cells, neural differentiation, neocortical neurons, disease modeling, autism spectrum disorders (ASD), cellular phenotype

## Abstract

Autism spectrum disorder (ASD) is an early-onset neurodevelopmental disorder characterized by deficits in social communication, and restricted and repetitive patterns of behavior. Despite its high prevalence, discovery of pathophysiological mechanisms underlying ASD has lagged due to a lack of appropriate model systems. Recent advances in induced pluripotent stem cell (iPSC) technology and neural differentiation techniques allow for detailed functional analyses of neurons generated from living individuals with ASD. Refinement of cortical neuron differentiation methods from iPSCs will enable mechanistic studies of specific neuronal subpopulations that may be preferentially impaired in ASD. In this review, we summarize recent accomplishments in differentiation of cortical neurons from human pluripotent stems cells and efforts to establish *in vitro* model systems to study ASD using personalized neurons.

## Introduction

Autism spectrum disorder (ASD) is a debilitating neurodevelopmental disorder characterized by impaired communication and social interactions, as well as restricted interests and repetitive behaviors (Devlin and Scherer, 2012). Approximately 1/50 children in North America are diagnosed with ASD, typically by the age of 3 years (Blumberg et al., 2013). The severity of symptoms varies greatly and the prevalence of intellectual disability, epilepsy, attention deficit/hyperactivity disorder, and obsessive-compulsive disorder is markedly higher in people with ASD than in unaffected individuals (Huguet et al., 2013). Despite the complexity and heterogeneity of ASD, genetic studies, post-mortem brain analyses, and functional imaging studies have resulted in the widely accepted hypothesis the ASD arises from dysfunctional neuronal communication in the neocortex (Zikopoulos and Barbas, 2013).

ASD is primarily viewed as a genetic disorder, although the genetic underpinnings of ASD are complex. Family and twin studies have revealed that the heritability of ASD is as high as 90%, but causal genomic variations have only been identified in ~25% of cases. These have mostly consisted of relatively rare genetic variations, none of which account for more than ~1% of ASD cases (Devlin and Scherer, 2012). To date, several dozen high priority ASD candidate genes have been identified, many of which encode proteins that localize to synapses [e.g., SH3 and multiple ankyrin repeat domains (SHANK) 2, SHANK3, Neuroligin (NLGN)-1, NLGN-3, NLGN-4X, Neurexin (NRXN)-1, and NRXN-3] and regulate their development, maturation, and function (Zoghbi and Bear, 2012). ASD-associated genomic variations can occur *de novo* in affected individuals. In familial cases, these variants are often inherited from unaffected parents, suggesting either incomplete penetrance or modifier genes. For example, four autistic individuals with *de novo SHANK2* mutations have additional genetic variations at ASD candidate loci, suggesting a “mutliple hit” model of ASD (Leblond et al., 2012; Chilian et al., 2013).

Mice engineered to encode human ASD-associated mutations often recapitulate behavioral hallmarks of the disorder and are readily amenable to experimental analyses (Silverman et al., 2010; Jiang and Ehlers, 2013). Many synapse-associated ASD candidate genes have been knocked-out in mice, revealing a wide range of synaptic phenotypes that may contribute to ASD. *Nlgn-1* knockout mice exhibited altered excitatory synaptic transmission (Blundell et al., 2010) and knockdown results in decreased cortical synapse numbers (Kwon et al., 2012). *Nrxn-1α* knockouts exhibit reduced spontaneous excitatory synaptic activity, with no change in inhibitory synapse function (Etherton et al., 2009). Mice with the ASD-associated *Nlgn-3* R451C mutation exhibit increased inhibitory neurotransmission in the cortex (Tabuchi et al., 2007; Etherton et al., 2011), but increased excitatory neurotransmission in the hippocampus (Etherton et al., 2011). Finally, knockouts of *Shank2* and *Shank3* support a role for SHANKs in excitatory synapse function, although distinct phenotypes were observed in different models (Durand et al., 2007; reviewed in Jiang and Ehlers, 2013). Unfortunately, mice with ASD-associated mutations rarely exhibit phenotypes unless these mutations are homozygous, which are exceptionally rare in people with ASD (Ey et al., 2011; Won et al., 2012). These findings suggest that heterozygous disruption of individual candidate genes may be necessary, but not sufficient for development of the disorder, and that other genetic variables may play a role (Huguet et al., 2013). An alternative explanation is that ASD candidate genes have slightly different functions in human neurons. Both of these limitations of mouse models can be overcome with the use of induced pluripotent stem (iPSC) technology, which allows the generation of personalized human neurons from people with ASD.

iPSCs represent an incredible new avenue for the modeling of ASD (Ross and Ellis, 2010). Donor-derived cells (e.g., dermal fibroblasts from a skin biopsy or peripheral blood mononuclear cells) are reprogrammed into iPSCs by forced expression of four pluripotency-associated transcription factors: OCT4, SOX2, KLF4, and c-MYC (Takahashi et al., 2007). Resultant iPSC lines exhibit functional properties of human embryonic stem cells (hESCs), including the ability to differentiate into any cell type in the human body. For experimental analyses, iPSCs provide an unlimited supply of ASD-specific neurons. To date, iPSC-derived neurons have been used to generate personalized neurons from individuals with neurodevelopmental disorders that include autistic features—RTT (Marchetto et al., 2010; Cheung et al., 2011), Timothy syndrome (TS) (Paşca et al., 2011), and Phelan McDermid syndrome (PMDS) (Shcheglovitov et al., 2013)—and have revealed disorder-specific neuronal phenotypes, including dysfunctional synaptic connectivity. However, this approach has yet to be applied to ASD as the fifth edition of the Diagnostic and Statistical Manual of Mental Disorders excludes individuals with syndromic neurodevelopmental disorders from an ASD diagnosis (American Psychiatric Association, 2013). Although iPSC-derived neurons have been generated from people with ASD, no functional experiments were described (DeRosa et al., 2012). As such, the potential of iPSC technology has yet to be fully applied to modeling ASD, although many groups are actively pursuing this approach.

The generation of iPSCs has become commonplace. However, efficient differentiation of these cells into specific neuronal subtypes remains challenging. As discussed above, one of the prevailing hypotheses suggest that ASD arises due to dysfunctional synaptic communication in the neocortex. Successful generation of ASD-specific cortical neurons will improve our understanding of how ASD develops and may allow for identification of novel therapeutics. In this review, we discuss (1) recent advances in technology of cortical differentiation from human pluripotent stem cells (hPSCs) based on the knowledge of *in vivo* cortical development, (2) recent findings from human iPSC (hiPSC)-based models of RTT, TS, and PMDS, and (3) future directions for optimization of cortical differentiation and modeling of ASD, as well as potential applications of this exciting technology.

## Development of the neocortex

A thorough understanding of neocortical development can inform methodology for cortical neuron differentiation from hPSCs and define neuronal characteristics that should be considered in validating the identity and functionality of resultant neurons. This is especially important for hPSC-based ASD modeling, as abnormal neocortical development has been directly associated with the etiology of some ASDs (Kwan, 2013). Thus, we first give an overview of neuronal composition in the neocortex and its origins, based on the studies of animal models.

The mammalian neocortex has a well-organized six-layered structure. Each cortical layer contains a characteristic distribution of neuronal cells with distinctive shape, size, and neurochemical and electrophysiological properties, which make local or long distance connections with other cortical region or subcortical compartments (Douglas and Martin, 2004; Migliore and Shepherd, 2005). Neurons in the neocortex can be broadly categorized into two types: excitatory projection neurons and inhibitory interneurons. Excitatory projection neurons, which comprise around 80% of the neocortical neuronal population, mainly originate from neuroepithelial cells of the germinal zone in the dorsal telencephalon (pallium) (Molyneaux et al., 2007). They have a characteristic pyramidal shape with a long apical dendrite, multiple basal dendritic branches with spines receiving signals from other neurons, and a long axon making synaptic connections via the excitatory neurotransmitter glutamate (Spruston, 2008). On the other hand, inhibitory interneurons develop and migrate from distinct progenitors of the germinal zone of the ventral telencephalon (subpallium), mostly from the medial ganglionic eminence (MGE) and caudal ganglionic eminence (CGE) (Wonders and Anderson, 2006). They make up the remaining 20% of cortical neurons and make local connections using the inhibitory neurotransmitter GABA. Inhibitory interneurons in the neocortex display an astonishing diversity with over 20 subtypes based on morphology, electrophysiological properties, and expression of calcium binding proteins and neuropeptides (Petilla Interneuron Nomenclature Group, 2008).

### Development of neocortical excitatory neurons

**Figure 1:**
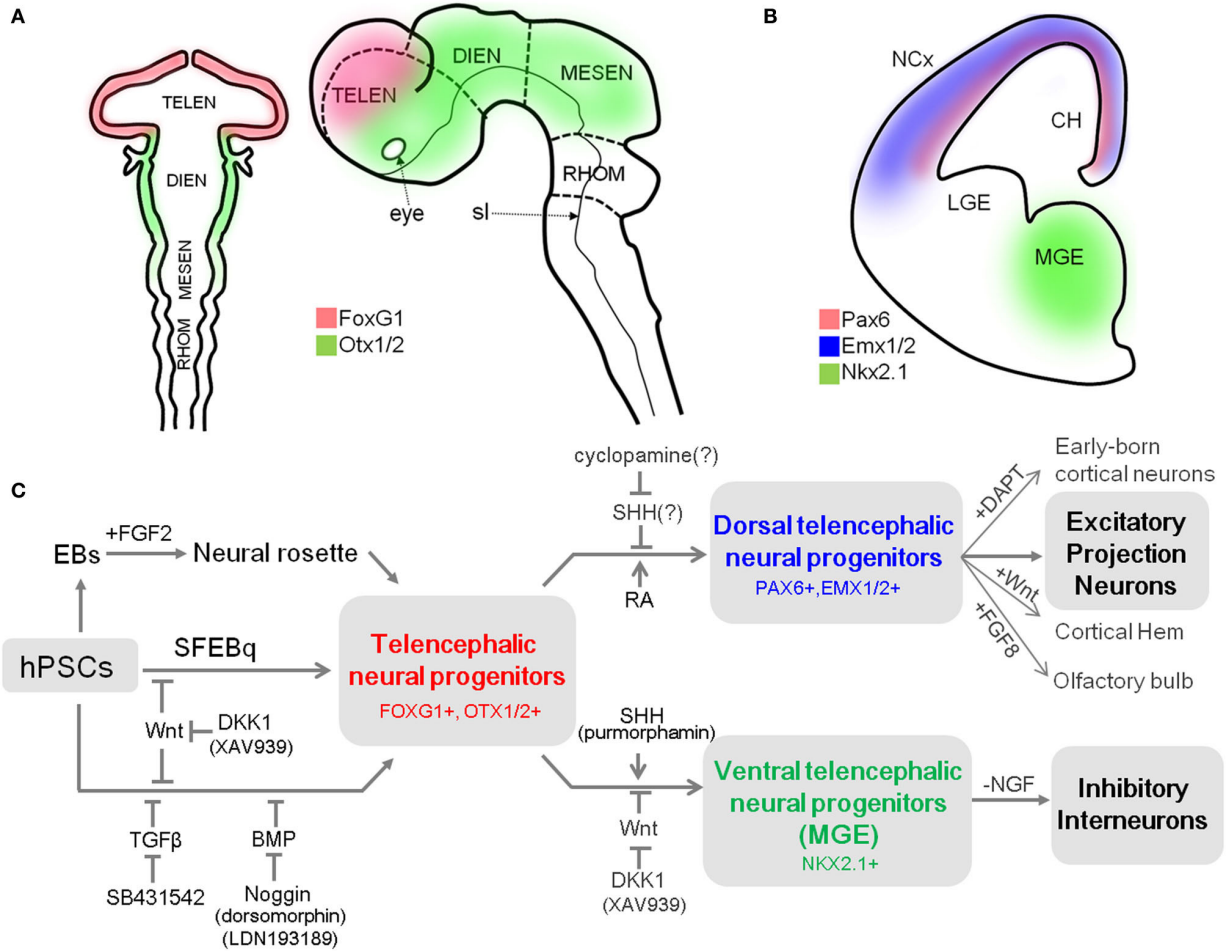
**A summary of differentiation from hPSCs into neocortical excitatory projection neurons and inhibitory interneurons. (A)** Schematic mouse brain at E8 (left) and at E10.5 (right) depicting the expression domain of Foxg1 and Otx1/2. **(B)** Coronal hemi-section view of mouse brain at E13 showing the distinctive expression domains of Emx1/2, Pax6, and Nkx2.1, abbreviation: TELEN, telencephalon; DIEN, diencephalon; MESEN, mesencephalon; RHOM, rhombencephalon; sl, sulcus limitans; NCx, neocortex; LGE, lateral ganglionic eminence; MGE, medial ganglionic eminence; CH, cortical hem. **(C)** Human PSCs are induced into telencephalic neural progenitors in three main ways: (1) culturing EBs in suspension and isolation of neural rosette cell from the subsequent adherent culture of EBs (Zhang et al., 2001), (2) SFEBq method (Eiraku et al., 2008), and (3) dual-SMAD inhibition method (Chambers et al., 2009). Telencephalic fate can be facilitated by inhibition of the Wnt pathway during neural induction (Eiraku et al., 2008; Maroof et al., 2013; Nicholas et al., 2013). Telencephalic neural progenitors can be specified either to dorsal fate by blockade of endogenous SHH signal (Vazin et al., 2013) or exogenous RA treatment (Shi et al., 2012), or to ventral fate by additional activation of SHH signal (Liu et al., 2013a; Maroof et al., 2013; Nicholas et al., 2013) combined with Wnt inhibition (Li et al., 2009). Dorsal telencephalic progenitors can generate a variety of excitatory projection neurons (Eiraku et al., 2008; Shi et al., 2012; Lancaster et al., 2013), and also be further specified into (1) early-born cortical neurons such as Reelin-positive Cajal-Retzius cells or CTIP2-positive deep layer neurons depending on timing of DAPT treatment; (2) cortical hem by exogenous Wnt; and (3) olfactory bulb by FGF8 treatment (Eiraku et al., 2008). In contrast, ventral telencephalic progenitors can differentiate into functional GABAergic inhibitory neurons by either withdrawal of NGF in the culture medium (Liu et al., 2013b) or by adjusting the temporal window for SHH treatment during the ventralization step (Maroof et al., 2013).

In the widely accepted model of vertebrate neural induction, the first emerging neuroectodermal cells in the neural plate develop an anterior fate characterized by expression of transcription factors such as forkhead box G1 (*Foxg1*, also known as brain factor 1, *Bf1*) or orthodenticle homoebox 1/2 (*Otx1/2*) (Stern, 2001; Hébert and Fishell, 2008) (Figure 1A). As neural induction proceeds, the cells that position in relatively posterior regions are influenced by patterning factors, such as Wnts and retinoic acid (RA), and are subsequently reprogrammed to a caudal fate. In contrast, the cells in the anterior part of neural plate are less influenced by caudalizing factors due to the endogenous expression of their antagonists [e.g., Dickkopf-related protein 1 (DKK1, a Wnt signal antagonist)], and maintain the acquired anterior character (Glinka et al., 1998; Wilson and Houart, 2004). Once the neural tube forms, the most anterior region rapidly expands to form the telencephalon, which is divided into two distinctive regions, the dorsal telencephalon and the ventral telencephalon by gradients of dorso-ventral patterning factors (Wilson and Rubenstein, 2000).

The pallial neural progenitors, the main source of neocortical projection neurons, are developed under the influence of Wnt and BMP signaling. They can be defined by the expression of a set of transcription factors, which includes Foxg1, paired box 6 (*Pax6*), empty spiracles homolog 1/2 (*Emx1/2*) in mice (Figures 1A,B) (Molyneaux et al., 2007). Mouse genetic studies have provided evidence that these transcription factors are responsible for the establishment and maintenance of neocortical progenitors and suppress alternative fates. For example, removal of *Foxg1* in the mouse embryo causes the absolute absence of neocortical progenitors, which eventually results in severe malformation of the neocortex (Xuan et al., 1995; Muzio and Mallamaci, 2005). In turn, *Pax6* is essential for proliferation of progenitors in the pallium (Estivill-Torrus et al., 2002), and its absence in the murine embryonic brain results in the expansion of a domain expressing ventral progenitor makers, suggesting that it is essential for the establishment and maintenance of pallial progenitors (Stoykova et al., 2000). Accordingly, the appropriate expression of these transcription factors in cortical progenitors is a prerequisite for their progressive specification to projection neurons. Their expression can be used as a reliable marker for dorsal telencephalic identity of the progenitor stage during neural differentiation of hPSCs.

Once neurogenesis begins, neuroepithelial cells in the dorsal telencephalon acquire features of neural stem cells known as radial glial cells (RGCs). Through asymmetric cell division, RGCs give rise to (1) self-renewed RGCs that remain in the ventricular zone (VZ) throughout corticogenesis, and (2) committed daughter cells that can migrate out (Kriegstein and Alvarez-Buylla, 2009). The committed daughter cells either become early-born cortical neurons or remain in a defined domain next to the VZ called the subventricular zone (SVZ), where they undergo cell division as intermediate progenitors to generate diverse cortical projection neurons across multiple neocortical layers (Götz and Huttner, 2005). Recent clonal analysis of progenitors in the SVZ of human cortex revealed the appearance of distinctive progenitors called outer radial glial cells (oRGCs) immediately outside the SVZ (Hansen et al., 2010). The diversity of the progenitor population in the human brains contributes to their structural complexity, and results in a vast increase in the number of projection neurons and overall volume of the neocortex relative to those of rodents and other carnivores (Lui et al., 2011).

In general, early-born projection neurons migrate out from the proliferative area settling in the deep layer first, and later-born projection neurons migrate beyond those in deeper layers to reach the upper layers. Such “inside-out” patterning of post-mitotic neurons in a spatio-temporally controlled manner accounts for the well-organized layered structure of neocortex (Rash and Grove, 2006). Recent studies in the mouse show that each subtype and laminar specification in the neocortex is programmed by expression of particular transcription factors in cortical progenitors and neurons (reviewed by Molyneaux et al., 2007; Kwan et al., 2012). These genes play essential roles in refining the specific molecular identity of each layer (neuronal migration and the proper positioning) (Alcamo et al., 2008; Chen et al., 2008), layer-dependent axonal connectivity (Han et al., 2011), and even dendritic arborization and spine morphology (Cubelos et al., 2010). In addition, many studies have suggested that alteration in the proper expression of cortical layer-specific genes is associated with human neurodevelopmental disorders, including ASD (reviewed by Kwan, 2013).

### Development of neocortical gabaergic interneuron

Unlike excitatory projection neurons, neocortical inhibitory neurons arise from progenitors in the subpallial region, where cells are under the influence of SHH. Progenitors in the MGE are characterized by expression of Nkx2 homeobox 1 (Nkx2.1, also known as thyroid transcription factor 1, TTF-1) (Figure 1B) and Foxg1, which are both regulated by SHH (Sussel et al., 1999; Gulacsi and Anderson, 2006). In particular, Nkx2.1 plays a pivotal role in the induction of neocortical GABAergic neurons. Mutation of Nkx2.1 in mice results in significant loss of parvalbumin (PV)- and somatostatin (STT)-positive GABAergic neurons in the cortex (Sussel et al., 1999). On the other hand, GS homeobox 2 (Gsx2) specifies progenitors in CGE, where SHH-independent calreticulin (CR)-expressing GABAergic neurons are derived (Xu et al., 2010).

A remarkable feature in the development of neocortical interneurons is that they—unlike projection neurons—undergo tangential migration from their place of origin to their cortical destination. Several genetic studies in humans and mice have implicated dysfunctional development or migration of GABAergic interneurons with many psychiatric and neurodevelopmental disorders (Powell et al., 2003; Gant et al., 2009; Poitras et al., 2010). Together, these data emphasize the critical role of GABAergic neurons in proper function of the neocortex.

## Current progress in cortical neuron derivation from hPSCs

Impairment of proper development and migration of both excitatory projection neurons and inhibitory interneurons in the neocortex contributes to neurodevelopmental disorders. Therefore, the ability to generate those neurons from hPSCs is a powerful approach for assessing their molecular and cellular phenotypes and essential mechanisms underlying disease onset. Currently, most protocols for cortical differentiation from hPSCs are based on a few core methods that were developed using hESCs (Table 1). Understanding how these methods work and the basic characteristics of neural progenitors they generate is critical for developing novel protocols for differentiation of specific subtypes of cortical neurons. Thus, we first introduce several methods that are most frequently used to generate neural progenitors from hPSCs. After that, we discuss recent accomplishments in differentiation of cortical excitatory projection and inhibitory neurons from hPSCs (summarized in Figure 1C).

**Table 1:** **Comparison among common methods for neural differentiation of hPSCs**.

	**Culture method**	**Strategy for neural differentiation**	**Regional identity of neural progenitors**	**Advantage**	**References**
EB formation-neural rosette isolation method	EB formation by lifting hPSC colonies and following adherent culture of EBs	Induction and isolation of neural rosettes without morphogens	Dorsal telen-diencephalon (PAX6+, OTX2+, FOXG1+)	Highly reproducible across many hPSC lines	Zhang et al., 2001; Pankratz et al., 2007
Dual-SMAD inhibition method	Adherent monolayer culture of dissociated hPSCs	Inhibition of BMP/ Nodal signals	Dorsal telen-diencephalon (PAX6+, OTX2+, FOXG1+)	Highly rapid and efficient	Chambers et al., 2009
SFEBq method	EB formation by re-aggregation of dissociated hPSCs	Inhibition of WNT/BMP/Nodal signals	Dorsal telencephalon (FOXG1+, EMX1+)	Suitable for cortical differentiation	Watanabe et al., 2005; Eiraku et al., 2008

### Neural differentiation from hPSCs

Zhang and colleagues published the first report on neural differentiation from human ESCs (Zhang et al., 2001). In their study, embryoid bodies (EBs) are generated by lifting hESC colonies and cultured in suspension devoid of mitogens for a short period of time. Next the EBs are grown in adherent culture in defined media containing N2 supplement and basic fibroblast growth factor (bFGF) and allowed to form “neural rosettes.” This unique cellular arrangement of epithelial cells is reminiscent of cross sections of the developing neural tube and is now considered a hallmark of successful neural induction. These cells extensively express many neural stem cell markers such as Nestin, Musashi-1, and polysialylated-neuronal cell adhesion molecule, vigorously proliferating in the presence of bFGF after enzymatic isolation, and generate neurons, astrocytes, and oligodendrocytes both *in vitro* and *in vivo* (Zhang et al., 2001). In a subsequent study, Zhang's group found that neural progenitors generated in this manner mainly exhibit the anterior identity even though no regional cues were used throughout the differentiation (Pankratz et al., 2007). The regional identity of hESC-derived neural progenitors appears to be convertible by patterning cues. Timely treatment with particular morphogens such as SHH and Wnts, or their agonists/antagonists redirects the regional identity of hESC-derived neural progenitors to either ventral or caudal fate. The fate plasticity of hESC-derived neural progenitors has led to the development of many methods for generating different neuronal subtypes, such as midbrain dopaminergic neurons (Yan et al., 2005), spinal motor neurons (Li et al., 2005), as well as cortical neurons (Li et al., 2009).

Another EB-like structure-based neural differentiation method was published by Sasai's group. Their first study described a serum-free EB-like protocol (which they called SFEB) to generate neurons from mouse ESCs (mESCs). Quantitative analysis revealed that around 80% of total cells were Sox1-positive neural lineage in 5 days. Substantial numbers of cells derived by SFEB culture express forebrain markers such as *Foxg1* and *Otx2*, although this number was still low (~20% of total cells) compared to the number in hESC differentiation (Watanabe et al., 2005; Pankratz et al., 2007). A key step in this protocol was the dissociation of mESCs to single cells to form EB-like structures of a defined size, and cultured in serum-free media. However, this protocol was difficult to adapt to hESCs, which are remarkably vulnerable to apoptosis upon dissociation (Ohgushi et al., 2010). To circumvent this problem, Sasai's group employed Rho-dependent protein kinase (ROCK) inhibitor, which promotes the survival of dissociated hESCs. With it, they successfully reproduced the SFEB method with hESCs (Watanabe et al., 2007). As was observed with mESCs, human neural cells differentiated by SFEB culture were frequently positive for FOXG1 (~32% of total cells), and could be patterned toward either ventral or dorsal fate. More recently, the same research group further optimized this method in terms of speed, efficiency, and reproducibility of neural conversion by quick re-aggregation of ESCs in round-bottom well-plates (Eiraku et al., 2008). In this manner, over 95% of total cells exhibited features of neuroepithelial cells at day 5 of differentiation. Most interestingly, the majority expressed dorsal telencephalic markers. Since this method exhibited a striking resemblance with *in vivo* corticogenesis and mainly generated cortical excitatory neurons, we will return to it in the next section.

Another approach that has been used to induce neural progenitors from ESCs was co-culturing with mouse stromal feeder cells that are known to have neural inducing activity (Kawasaki et al., 2000; Elkabetz et al., 2008). Despite the method's robustness, the involvement of non-human cells and the requirement of relatively long period of time for neural induction (>3 weeks) made this method less attractive for biomedical applications.

Recently, Studer's group reported a remarkably simple and robust method for neural induction of hESCs (Chambers et al., 2009). In adherent single cell-culture of hESCs under serum-free conditions, simultaneous modulation of endogenous BMP and Activin/Nodal signaling by treatment with Noggin (BMP inhibitor) and SB431542 (Activin/Nodal inhibitor) converted hESCs to largely PAX6-positive neuroectodermal cells competent to form neural rosettes in 11 days of differentiation. Since each signaling pathway recruits SMAD proteins as intracellular signal transducers, this was often referred to as the dual-SMAD inhibition approach. Interestingly, most neural cells generated by this method express FOXG1 and OTX2, along with robust expression of PAX6, suggesting dorsal telencephalic identity (Chambers et al., 2009). The feasibility and robustness of this method has resulted in its relative popularity in the field, as it provides highly enriched neural precursors for disease modeling (Lee et al., 2009).

Given that hESC-derived neural progenitors from different research groups exhibit regional identity of the dorsal telencephalon, hPSCs are likely to have an innate program for differentiation into neural cells found in this brain region regardless of method (Pankratz et al., 2007; Elkabetz et al., 2008; Chambers et al., 2009). This seems consistent with the theory that the first neural precursors generated during vertebrate neural induction acquire dorsal telencephalic identity by default (Muñoz-Sanjuán and Brivanlou, 2002). However, current protocols for neural differentiation were developed and tested with only a few widely-used cell lines (e.g., H9). Moreover, a recent report argued that neural progenitors generated from different hESC lines differ in regional identity when derived by the same protocol, potentially due to differences in epigenetic programming (Wu et al., 2007). The assumption hPSC lines all follow a default pathway to a dorsal telencephalic identity may be a hasty generalization. Thus, we recommend determination of the regional identity of neural progenitors from new hPSC lines before further neuronal specification.

### Generation of excitatory projection neurons from hPSCs

Sasai's group pioneered directed cortical differentiation from both mouse and human ESC by SFEB method and regional patterning. They optimized the previous SFEB method by allowing a defined number of cells to re-aggregate quickly in round-bottom 96-well plates under the influence of several regionalizing factors (referred to as the SFEBq method). This remarkably improved the differentiation efficiency of mESCs to dorsal telencephalic neural precursors, evidenced by expression of *Foxg1* (~65–75% of total cells) and *Emx1* (~89% of *Foxg1*-positive cells) (Eiraku et al., 2008). Interestingly, this system generated self-organized cellular aggregates of cortical progenitors and cortical neurons from mESC in the spatio-temporal manner reminiscent of *in vivo* corticogenesis. SFEBq-induced cortical progenitors even respond to cues directing regional pallial induction, such as FGF, which refines the pallial fate along the rostro-caudal axis, and BMP/Wnt, which induces expression of choroid flexus or cortical hem markers (Eiraku et al., 2008). However, well-organized laminar formation of cortical neurons did not appear within SFEBq-induced mouse cortical tissues, and hESCs failed to generate neurons of upper cortical layers in this system.

Upon further refinement, SFEBq approaches have been successfully applied to hPSCs. Vaccarino and colleagues reproduced this approach by generating hiPSC-derived multilayered cortical structures, which predominantly exhibited the gene expression profile of dorsal telencephalon (Mariani et al., 2012). More recently, Knoblich and colleagues developed an advanced *in vitro* differentiation method adapting the SFEBq system by culturing matrigel-embedded EBs in a spinning bioreactor (Lancaster et al., 2013). This system succeeded in establishing a cerebral organoid culture system, which reproduces many features of human cortical development in a more precise manner. In particular, they found characteristic progenitor zone organization, including abundant RGC and oRGC populations, with ventral telencephalic progenitors migrating into a cortical layer-like structure. Moreover, the ability to produce mature cortical neuron subtypes in an “inside-out laminar pattern” was unique in recapitulating *in vivo* corticogenesis not observed with the original SFEBq method. Most interestingly, cerebral organoids from hiPSCs with a CDK5RAP2 mutation, which causes microcephaly in humans, resulted in smaller neural tissues with impaired progenitor regions, which has never before been recapitulated in animal models (Lancaster et al., 2013). Most recently, Sasai's group optimized their SFEBq method by culturing cell aggregates in enriched medium and high oxygen (40%), thereby generating a three-dimensional neuronal mass with features resembling human fetal cortex in the early second trimester. Their new method surpassed the limitations of their previous method and achieved axial polarity, human specific oRGC populations, and “inside-out” laminar structure of cortical neurons (Kadoshima et al., 2013). By providing a robust methodology for efficient generation of cortical neurons from hPSCs, these three-dimensional differentiation approaches represent a powerful tool for investigation of human brain development and neurodevelopmental disorders. The potential to characterize electrophysiological properties, function, and connectivity of targeted neuronal populations organized in a multi-layered cortical pattern is of great utility for the study of ASD.

In contrast to the three-dimensional differentiation system, the adherent monolayer-differentiation system may provide a more feasible tool to examine morphology and synaptic connectivity, which are of interest as the main cellular phenotype of ASD neurons. It can also be scaled-up for drug screening platforms. Livesey and colleagues described a defined cortical differentiation condition by employing the monolayer culture and dual-SMAD inhibition (Shi et al., 2012). Interestingly, they found that RA was an essential factor for robust differentiation of cortical progenitors with PAX6 and OTX1/2-immunoreactivity. Cortical progenitors generated by their method displayed neural rosette structures with the apico-basal polarity and characteristic interkinetic nuclear migration during cell division. More importantly, this method recapitulated complex human progenitor populations including intermediate progenitors and oRGCs with unipolar basal processes, as seen in the developing human brain. In addition, birth-dating analysis using BrdU labeling revealed the appearance of both deep-layer and upper-layer cortical neurons in a temporal manner, paralleling *in vivo* corticogenesis over 90 days of neuronal maturation (Shi et al., 2012). With this protocol, the same group generated cortical neurons derived from Down syndrome (DS)-specific iPSCs. These neurons exhibited pathological features of early-onset Alzheimer's disease seen in DS patients, demonstrating the applicability of this protocol for modeling cortical disease (Shi et al., 2013). Although the role of RA as a modulator for cortical differentiation needs further mechanistic characterization, this study was the first to recapitulate the diversity of cortical progenitors and generation of cortical subtypes from hPSCs in a temporally-controlled manner.

There have also been attempts to obtain cortical projection neurons by inhibiting cellular signal(s) that drive alternative fates. Since the cerebral cortex develops in the dorsal telencephalic region of the embryonic brain, blockade of intrinsic ventralizing and/or caudalizing signals during neural induction of ESCs may lead to neural precursors with dorsal telencephalic fate. Vanderhaeghen's group was the first to test this hypothesis in mESCs (Gaspard et al., 2008). They found that a low density culture of mESCs in chemically defined media devoid of any regional cues generated Otx1/2-positive neural progenitors, many of which were co-labeled with Nkx2.1. Therefore, at least in the mESC system, the default differentiation condition may favorably generate ventralized telencephalic progenitors, possibly because of high endogenous Shh levels. As support for this hypothesis, the same group showed that the inhibition of intrinsic Shh signaling by treatment with cyclopamine, a small molecule inhibitor of Shh signal, abolished ventral marker expression in neural progenitors, whereas it largely elevated dorsal marker expression. Cortical progenitors differentiated in this manner could mainly differentiate into functional excitatory neurons with pyramidal shape that expressed a series of transcription factors corresponding to each cortical layer in a temporal manner reminiscent of *in vivo* corticogenesis (Gaspard et al., 2008).

Unlike those in the mESC system, neural progenitors derived from hESCs tend to retain dorsal telencephalic fate in many cases, as discussed above. The difference in dorso-ventral patterning between these systems may be explained by distinctive intracellular programming. While endogenous Shh signal dominates during early neural induction of mESCs (Gaspard et al., 2008), Zhang and colleagues found that Wnt signaling prevails during neural induction of hESCs. In addition, they showed that Wnt inhibition facilitated the ventralization of neural progenitors by exogenous SHH, supporting the idea that the endogenous Wnt signaling underlies the differentiation inclination of hPSC toward dorsal fate (Li et al., 2009). Consistent with this, a recent study from Vanderhaeghen's group showed that cyclopamine treatment was not required for induction of the dorsal telencephalic fate in the hPSC system (Espuny-Camacho et al., 2013). However, Schaffer and colleagues recently showed that SHH inhibition by cyclopamine was necessary to generate excitatory neurons expressing cortical markers from hPSCs (Vazin et al., 2013). Thus far, the involvement of SHH signaling in the induction of dorsal telencephalic fate of hPSC-derived neurons is controversial and needs further study.

Ghosh and colleagues suggested a procedure for efficient differentiation of forebrain-type neurons via aggregate formation in multi-well plates in the presence of Noggin (Kim et al., 2011a). After adherent culture of aggregates on matrigel for a few days, most colonies developed neural rosettes that highly expressed transcripts of several dorsal telencephalic markers, such as *SOX1, PAX6, SIX3*, and *EMX2*. Continuous treatment with Noggin seemed critical for maintaining rosette structure and inducing telencephalic fate, and SHH-inhibition by cyclopamine did not facilitate the acquisition of dorsal fate. Further differentiation by dissociating neural rosette cells and coculturing them with rat astrocytes generated functional excitatory neurons. This study also assessed synaptic dysfunction by employing an artificial synapse formation assay, in which hPSC-derived neurons were co-cultured with HEK293T cells that expressed either normal or mutant types of *NLGN-3* and *NLGN-4*. In this system, hPSC-derived neurons were able to form presynaptic specializations on the HEK293T cells that expressed wild-type NLGNs more efficiently than on those that expressed ASD-associated mutant NLGNs (Kim et al., 2011a). This study was a practical example of an efficient cortical differentiation method combined with an assay of synapse formation to assess the functional impact of ASD-associated mutations.

In recent years, several studies have provided multiple methods for generating cortical excitatory neurons from hPSCs that recapitulate *in vivo* corticogenesis and even human-specific features not seen in animal models. Although *in vitro* modeling ASD using cortical differentiation technology is still in its infancy, it is becoming clear that the current accomplishments already provide robust models for investigating cellular phenotypes that are directly relevant to ASD pathophysiology.

### Differentiation of neocortical inhibitory neurons from hPSCs

In recent years, many studies of autistic people and ASD animal models have strongly implicated dysfunction of the GABAergic system in the pathophysiology of ASD (reviewed by Chattopadhyaya and Cristo, 2012). Perturbation of subtle excitatory-inhibitory balance due to loss or dysfunction of GABAergic interneurons can lead to hyperexcitability and/or impaired cortical oscillations, thereby resulting in various psychiatric and neurodevelopmental disorders. Given that epilepsy is more prevalent in children with ASD (Viscidi et al., 2013) and epileptiform activity in the prefrontal cortex is associated with deficits in social interaction (Hernan et al., 2013), dysfunction of the GABAergic system may be an especially important mechanism of ASD pathophysiology. Therefore, the ability to efficiently generate human cortical interneurons from people with ASD could serve as a valuable tool for investigating GABAergic system dysfunction in ASD pathophysiology, as well as facilitating drug discovery. Here, we summarize recent results in obtaining GABAergic interneurons from hPSCs.

Zhang and colleagues obtained human neuroepithelial cells predominantly expressing PAX6 around 8–15 days of neural induction. This was achieved using the EB formation-neural rosette isolation method without exogenous morphogens, which exploits the default telencephalic specification of hESCs (Liu et al., 2013a). By exposing those cells to high doses of SHH (over 500 ng/ml) or purmorphamine (1.5 μM), a small molecule agonist of SHH signaling, they succeeded in generating MGE-like neural progenitors, mainly characterized by expression of NKX2.1, and abolished PAX6 and EMX1-positive dorsal telencephalon and MEIS1/2-positive lateral ganglionic eminence population. Neuronal maturation of NKX2.1-positve cells on hESC-derived astrocytes in the presence of nerve growth factor (NGF) gave rise to both functional choline acetyl-transferase-positive basal forebrain cholinergic neurons and GABAergic neurons in similar proportions, faithfully recapitulating *in vivo* differentiation from MGE precursors (Liu et al., 2013a). Interestingly, they also found that depletion of NGF, a simple modification, favored GABAergic differentiation with a purity of over 90% in the same conditions (Liu et al., 2013b).

Two different groups sought a direct way to pattern hPSC-derived neural precursors into cortical GABAergic interneurons. Specifically, they directed telencephalic fate prior to subsequent ventralization for differentiation, instead of depending on spontaneous telencephalic specification. Studer and colleagues described a robust pharmacological method that allows efficient modulation of signals implicated in neural patterning. In particular, they inhibited endogenous Wnt signaling to facilitate telencephalic differentiation (Maroof et al., 2013), inspired by previous findings that Wnt can suppress forebrain induction in several vertebrates (Yamaguchi, 2001; Nordström et al., 2002). Treatment with XAV939, a small molecule inhibitor of the canonical Wnt pathway, during neural induction through dual-SMAD inhibition significantly increased the proportion of neural progenitors expressing FOXG1. In subsequent dorso-ventral patterning, activation of SHH signaling by the treatment of purmorphamine in a specific temporal window (day 6–18) was efficient for robust co-induction of NKX2.1 with FOXG1. Interestingly, fine temporal tuning of SHH signal activation (day 10–18) even discriminated between different subtypes of ventral progenitors, those co-expressing OLIG2 with NKX2.1 and FOXG1, and telencephalic GABAergic neurons expressing SST or PV after further differentiation. Such robustness makes this method more attractive for future investigations of specific roles for interneuron subtypes in the pathophysiology of neuropsychiatric disorders (Maroof et al., 2013).

Kriegstein and colleagues took a similar approach to enrich for neural progenitors with a telencephalic ventral fate from hPSCs (Nicholas et al., 2013). In this study, they exposed *NKX2.1::GFP* knockin reporter hESCs to DKK1 and purmorphamine under the combination of the SFEBq method (Eiraku et al., 2008), and the EB formation-neural rosette isolation method (Zhang et al., 2001). As a result, about 90% of differentiated cells were positive for GFP, 81.5% of which co-expressed FOXG1 at particular temporal conditions of DKK1 (for initial 15 days) and purmorphamine treatment (for initial 35 days). Co-expression of OLIG2 and MASH1 at the neural progenitor stage, and doublecortin or GABA immunoreactivity after further differentiation supported their MGE-like identity. Further differentiation after cell sorting GFP-positive cells efficiently generated multiple subtypes of functional forebrain GABAergic neurons both *in vitro* and *in vivo* (Nicholas et al., 2013).

Recently, another approach was developed for generation of cortical interneurons from the CGE. In contrast to STT and PV-expressing GABAergic neurons, which mostly originate from the MGE, the developmental mechanism of calreticulin (CR)-type interneurons that arise mostly in the CGE was not well-known. Rodríguez and colleagues illustrated that activation of Activin signaling facilitated the induction of CGE identity during neural differentiation of mouse and human ESCs, and enriched for CR-expressing GABAergic neurons (Cambray et al., 2012). Given the implication of CR-expressing interneurons in cases of epilepsy (Tóth et al., 2010), this approach may also be used for investigating impairment of the inhibitory system in people with ASD.

Despite differences in the details of differentiation methods, the studies described above showed that strong SHH signaling promotes the ventralization of telencephalic progenitors and generates MGE-like neocortical GABAergic interneurons (Sousa and Fishell, 2010). More importantly, each approach presented not only efficient methodologies for generating neocortical GABAergic interneurons, but also provided new insights into developmental mechanisms of these cells, which not been observed in previous mouse studies. Thus, current advances in the development of neocortical interneurons from hPSCs are promising for elucidating the role of inhibitory interneurons in the etiology of ASD.

## Deriving neurons from hPSCs to model neurodevelopmental disorders

Several research groups have recently used hPSCs to model neurodevelopmental disorders that include autistic features, such as Rett syndrome (RTT) (Marchetto et al., 2010; Ananiev et al., 2011; Cheung et al., 2011; Kim et al., 2011b; Li et al., 2013), Fragile X-syndrome (Urbach et al., 2010; Sheridan et al., 2011; Bar-Nur et al., 2012; Liu et al., 2012), Prader-Willi/Angelman syndrome (Chamberlain et al., 2010; Yang et al., 2010), Timothy syndrome (Paşca et al., 2011; Krey et al., 2013), and Phelan-McDermid syndrome (Shcheglovitov et al., 2013). Most of the studies obtained mature neurons by employing existing neural differentiation protocols and showed that neurons differentiated from affected individuals or from genetically modified hPSCs exhibited disease-related phenotypes (summarized in Table 2). Here, we discuss a few accomplishments in *in vitro* modeling for these disorder using iPSCs, and discuss the efforts to make effective and meaningful iPSC-based models of ASD.

**Table 2:** **A summary of neural differentiation methods and cellular phenotypes in current iPSC models for ASD-related syndromes**.

**Disorder (Genetic defects)**	**Differentiation method**	**Regional induction**	**Neuronal subtype**	**Neural phenotypes**	**Cellular phenotypes of mutant neurons**	**References**
RTT (*MeCP2*)	NPCs: EB formation-neural rosette isolation method Neurons^*^: Culturing EBs (without RA for 1 week and with RA for following 3 weeks) and plating them after dissociation	N/A	Excitatory neuron	TUJ1, MAP2, VGLUT1,	Fewer synapses, Reduced spine density, Smaller soma size, Altered calcium signal, Electrophysiological defect Reduced synaptic density was restored by treatment of IGF1 or gentamycin	Marchetto et al., 2010
RTT (*MeCP2*)	EB formation-neural rosette isolation method	N/A	N/D	MAP2	Smaller soma size	Cheung et al., 2011
RTT (*MeCP2*)	EB formation-neural rosette isolation method	N/A	N/D	Nestin, TUJ1	Defect in neuronal maturation	Kim et al., 2011b
RTT (*MeCP2*)	EB formation-neural rosette isolation method	N/A	N/D	TUJ1	Smaller nuclear size	Ananiev et al., 2011
Atypical RTT (*CDKL5*)	EB formation-neural rosette isolation method	N/A	Cortical excitatory neurons	TUJ1, MAP2, VGLUT1, CTIP2	Reduced number of synaptic puncta Lengthy spine protrusion	Ricciardi et al., 2012
RTT (*MeCP2*^†^)	Dual-SMAD inhibition in adherent culture	N/A	Excitatory neurons	MAP2, TUJ1, VGLUT1	Smaller soma/nuclear size Reduced dendritic complexity, Electrophysiological deficits Global reduction in transcription Impaired AKT/mTOR activity Mitochondria deficit	Li et al., 2013
TS (*CACNA1C*)	EB formation-neural rosette isolation method	N/A	Cortical neurons	46 neural/neuronal markers were assessed by Fluidigm array	Defects in calcium-channel function Altered activity-dependent gene-expression/dendritic retraction Abnormality of lower cortical layer and callosal projection differentiation Abnormal catecholaminergic differentiation	Paşca et al., 2011; Krey et al., 2013
FXS (*FMR1*)	Manual isolation of neural rosette cells or isolation of PSA-NCAM-positive cells by MACS from the spontaneously differentiating iPSCs	N/A	N/D	TUJ1, GFAP	Fewer and shorter processes	Sheridan et al., 2011
FXTAS (*FMR1*)	EB formation-neural rosette isolation method accompanied by dual-SMAD inhibition	N/A	Excitatory neurons	MAP2, VGLUT1,	Shorter neurite length Fewer PSD95-positive synaptic puncta Sustained calcium response after glutamate application	Liu et al., 2012
AS (deletion in maternal chromosome 15q11-q13) PWS (deletion in paternal chromosome 15q11-q13)	EB formation-neural rosette isolation method	N/A	N/D	TUJ1	Phenotypic impairment was not specified	Chamberlain et al., 2010
PWS (translocation in maternal 15q11 and 4q27)	EB formation-neural rosette isolation method	N/A	N/D	MAP2, TUJ1	Phenotypic impairment was not specified	Yang et al., 2010
ASD (*NRXN1*)^‡^	EB formation-neural rosette isolation method	N/A	N/D	TUJ1, GFAP, and Global transcript-tome alteration was monitored by RNA-seq and Q-PCR	Reduced glial differentiation Altered gene expression related to cell adhesion and neuron differentiation	Zeng et al., 2013
PMDS (deletion in chromosome 22q13)	Dual-SMAD inhibition in adherent culture	Dorsal forebrain	Cortical neurons (both excitatory and inhibitory neurons)	MAP2, CaMKIIa, TBR1, CTIP2, SATB2, GAD67, and 66 sets of neural/neuronal marker expression were assessed by Fluidigm array	Impaired excitatory (both AMPA and NMDA-mediated) but not inhibitory synaptic transmission mainly due to loss of function of SHANK3 Reintroduction of SHANK3 and IGF1 application restore excitatory synaptic transmission	Shcheglovitov et al., 2013

* *In this study, the authors used different methods to derive NPCs and neurons, respectively*.

† *This study used genetically modified hESCs by deletion of exon 3 in the MECP2 locus by TALEN-mediated targeting*.

*Abbreviations: NPC, neural progenitor cells; EBs, embryoid bodies; N/A, not applicable; N/D, not defined; VGLUT1, vesicular glutamate transporter1; MAP2, microtubule-associated protein2; IGF1, insulin-like growth factor1, TUJ1, neuron-specific class III β-tubulin; mTOR, mammalian target of rapamycin; MACS, magnetic-activated cell sorting; GFAP, glial fibrillary acidic protein, CaMKIIa, calcium/calmodulin-dependent protein kinase IIa; GAD67, glutamic acid decarboxylase 67; TBR1; T-box brain 1; CTIP2, COUP-TF interacting protein2; SATB2, special AT-rich sequence-binding protein2; AMPA, α-amino-3-hydroxy-5-methyl-4-isoxazolepropionic acid; NMDA, N-methyl-D-aspartic acid*.

‡ *Small-hairpin RNA was used for knocking-down NRXN1 in hiPSC-derived neural stem cells*.

RTT is a severe neurodevelopmental disorder caused primarily by mutations in the X-linked gene *MECP2* (Methyl CpG-binding protein 2) (Chahrour and Zoghbi, 2007). Muotri and colleagues provided the first example of *in vitro* modeling of RTT by establishing iPSCs from individuals with various mutations in *MECP2*. They found that neural precursors derived from RTT-iPSCs did not show a distinct impairment in differentiation, proliferation, or survival. In contrast, RTT-neurons had fewer synapses, smaller soma size, and showed deficits in both calcium signaling and spontaneous excitatory synaptic communication compared to unaffected control neurons. Furthermore, they showed that some disease-related phenotypes (e.g., synaptic density) could be partially reversed by insulin-like growth factor 1 (IGF1) or gentamycin treatment, providing proof-of-principle evidence for the application of RTT-patient derived neurons for drug discovery (Marchetto et al., 2010). Importantly, smaller soma and nuclei have been repeatedly observed in RTT-iPSC derived neurons established by other research groups, regardless of the mutation or differentiation methods (Marchetto et al., 2010; Cheung et al., 2011; Li et al., 2013), suggesting that this phenotype might be a possible biomarker for future biomedical applications.

More recently, Jaenisch and colleagues established hESC lines with *MECP2* mutations using TALEN-mediated gene editing. By comparing mutant neurons to isogenic neurons from the parental hESCs, they investigated key molecular and cellular features of RTT (Li et al., 2013). MAP2-positive neuronal cells differentiated by the dual SMAD-inhibition method were mainly comprised of VGluT1-positive excitatory neurons, and displayed many typical deficits of RTT neurons previously shown in mouse models and neurons from RTT-specific iPSCs, such as smaller soma and nuclei, reduced neurite complexity, and electrophysiological deficits. Beyond this, they also detected a global translational impairment due to reduced AKT/mTOR activity, mitochondrial defects, an absence in activity-dependent gene transcription in hESC-derived neurons that lacked MECP2, which had not been observed previously in *in vivo* and *in vitro* models (Li et al., 2013).

Individuals with mutation of the cyclin-dependent kinase-like 5 (*CDKL5*) gene present with clinical features similar to RTT (Tao et al., 2004; Weaving et al., 2004). However, the mechanism underlying RTT-like symptoms caused by *CDKL5* mutations is largely unknown. Broccoli and colleagues addressed the function of *Cdkl5* in mouse hippocampal neurons by short-hairpin RNA-mediated knock-down of *Cdkl5*. These experiments showed that this *Cdkl5* is essential for proper dendritic spine structure and for activity of excitatory synapses by stimulating the phosphorylation-dependent interaction between NGL-1 (netrin-G1 ligand) and PSD95 (Ricciardi et al., 2012). They validated their finding in human neurons by generating iPSC lines from two individuals with *CDKL5* mutations and differentiating them into cortical neurons. Indeed, human neurons with a defective *CDKL5* gene had reduced numbers of synapses and long dendritic protrusions, as seen in mouse hippocampal neurons with knock-down of *Cdkl5*. Although the proposed mechanism was not fully addressed in human neurons, evidence from iPSC-modeling supports that the functional defect due to loss of *CDKL5* in affected individual results in disease-related phenotypes similar to RTT.

Many individuals with TS, caused by mutations in the L-type calcium channel *CACNA1C* gene, display features of ASDs (Splawski et al., 2004). Recently, Dolmetsch and colleagues established iPSC lines from individuals with TS and explored potential abnormalities in neuronal development or function (Paşca et al., 2011). iPSC-derived neurons with TS mutations had altered electrophysiological properties and activity-dependent gene expression, mainly resulting from aberrant calcium signaling. Interestingly, comparison of single-cell gene expression array profiles revealed reduced numbers of deep layer neurons expressing SATB2 in TS neurons compared to control neurons. This finding was confirmed in the brains of transgenic mice carrying mutation associated with type-1 TS. Since SATB2 is a critical transcription factor for development of callosal projection neurons (Alcamo et al., 2008), this finding strongly supported the idea that autistic symptoms seen in TS patients result from defects in cortical connectivity through the corpus callosum. In addition, the authors observed an abnormal increase in tyrosine hydroxylase-expression, consistent with the idea that altered synthesis of catecholamine may underlie ASD pathophysiology (D'Souza et al., 2009). In a follow-up study, both rodent cortical neurons with TS mutations and human neurons derived from TS-iPSCs exhibited activity-dependent dendritic retraction, which was caused by erroneous regulation of RhoA signaling by the mutated calcium channel (Krey et al., 2013).

Fragile X syndrome (FXS) is the most commonly inherited mental impairment, and is caused by expansion of CGG-repeats in the 5′ untranslated region of the fragile X mental retardation 1 (*FMR1*) gene, which leads to silencing of FMR1 expression. While Benvenisty and colleagues were the first to report the establishment of iPSC lines from FXS patients (Urbach et al., 2010), the first phenotypes of neurons derived from FXS-iPSCs were reported by Haggarty and colleagues, who showed that FXS-iPSCs preferentially generated Tuj1-positive neurons with shorter and fewer processes and more compact astrocytes (Sheridan et al., 2011). More recently, Hagerman and colleagues established isogenic pairs of iPSC lines from individuals with the related disorder fragile X-associated tremor ataxia syndrome (FXTAS) (Liu et al., 2012). iPSC-derived FXTAS neurons exhibited altered synapse formation, possibly caused by aberrant calcium currents. Interestingly, the mutant neurons exhibited a sustained calcium elevation after glutamate application, implying that enhanced type-I metabotropic glutamate activity may result in the imbalance of excitatory-inhibitory neuronal transmission (Liu et al., 2012).

AS and PWS are neurogenetic disorders caused by disruption of genes in imprinted regions of chromosome 15q11-13 (Ramocki and Zoghbi, 2008). AS results from loss of the maternal copy of the gene UBE3A, while the imprinted paternal gene is silenced; conversely, PWS results from loss of paternal genes (including the HBII-85 small nucleolar RNA cluster) and imprinting of maternal allele. Individuals with AS or PWS frequently exhibit intellectual disability, autism, severe seizures, and unusual or problematic behavior (Cassidy et al., 2012; Dagli et al., 2012). Chamberlain and colleagues provided the first example of disease modeling of AS and PWS and found that AS- and PWS-iPSCs retained the appropriate DNA methylation patterns. During neuronal differentiation, AS-iPSCs specifically repressed the paternal copy of *UBE3A*, concomitant with upregulation of UBE3A antisense transcripts, which is only expressed in neurons (Chamberlain et al., 2010). Similarly, Esteban and colleagues observed that iPSCs derived from individuals with PWS mutations bear an intact imprinting signature on the maternal allele, as seen in fibroblasts from which they originated (Yang et al., 2010). Although functional differences between affected neurons and normal neurons were not clearly addressed, these studies proved that iPSC-disease modeling of neurodevelopmental disorders of genomic imprinting is applicable.

Studies of hPSCs have also examined the function of ASD candidate genes. Wang and colleagues recently addressed the functional role of NRXN-1, a presynaptic protein of which mutation is highly associated with ASD pathogenesis, during the neurodevelopment of hPSC by functional knockdown. This study showed that reduction of NRXN-1 expression in hPSC-derived neural stem cells alters expression of many genes for the cell adhesion pathway (20 genes) and neuronal differentiation pathway (13 genes) with impairment of astrocyte differentiation, suggesting its functional impact on human neurodevelopment (Zeng et al., 2013). Dolmetsch and colleagues recently reported *in vitro* modeling of a rare neurodevelopmental disorder, Phelan-McDermid syndrome (PMDS), by generating iPSC lines from individual with heterozygous deletion of chromosomal locus 22q13.3 (Shcheglovitov et al., 2013). This locus includes the *SHANK3* gene, which is also mutated in ASD (Durand et al., 2007; Phelan and McDermid, 2012). In this study, the authors illustrated that *SHANK3* mutation causes important physiological defects in PMDS neurons, such as an imbalance of excitatory and inhibitory transmission due to impaired excitatory synapses. Importantly, they also found that PMDS neuronal phenotypes could be reversed by SHANK3 overexpression or treatment with IGF1.

These early studies highlight the remarkable promise of using personalized stem cell-derived neurons to investigate mechanisms underlying ASD pathophysiology. Even without aiming to generate specific neuronal subtypes, these experiments demonstrated deficits in neuronal specification (Paşca et al., 2011), synapse formation (Marchetto et al., 2010; Shcheglovitov et al., 2013), and excitatory neurotransmission (Shcheglovitov et al., 2013) in distinct ASD-related syndromes. However, an important consideration for most studies is the maturation status of iPSC-derived neurons. Neuronal age typically varies from 2 weeks to 3 months, with considerable variation in differentiation protocols and culture conditions. Furthermore, few markers are used to assess neuronal regional specificity, expression of ion channels, and neurotransmitter receptors. While single-cell expression profiling (using a platform like Fluidigm) can provide a snapshot of these characteristics (Paşca et al., 2011), it is currently limited to a fraction of the transcriptome and is relatively costly. Transcriptome profiling can overcome this drawback at the expense of single-cell resolution. For example, Vaccarino and colleagues (Mariani et al., 2012) used genome-wide expression microarrays to compare hPSC-derived cortical neurons to the developing human brain; these experiments revealed remarkable similarity between these neurons and the human frontal cortex at 8–10 weeks post-conception. Comparative expression analyses between cortical neurons derived from ASD-iPSCs and control-iPSCs could generate hypotheses regarding differences in the maturity, the functionality (for example, by expression changes of neurotransmitter receptors), and even regional identity of differentiated cortical neurons.

To date, most iPSC-based studies of neurodevelopmental disorders have been restricted to recapitulating the cellular phenotypes that were previously observed in animal models and postmortem examinations. To inform iPSC-based disease modeling, studies should aim to complement and extend this knowledge. A recent transcriptome analysis of postmortem brain tissues between individuals with ASD and control individuals identified 444 differentially expressed genes, and revealed the alteration of two distinct gene-expression modules related to synaptic communication and immune induction (Voineagu et al., 2011). Given that these features were observed in the postmortem brain, comparative transcriptomic analyses between neurons derived from ASD-iPSCs and control-iPSCs could highlight difference in gene expression during the development and progression of disease. To complement transcriptome-wide studies, comparative analyses of protein-protein interactions (the protein interactome) between ASD and control neurons may reveal alterations in normal cellular mechanisms. Considering the heterogeneity in ASD presentation and the underlying genetic lesions, multifaceted approaches with customized neurons will greatly improve our understanding of molecular mechanisms of ASD. By identifying the mechanistic pathways involved in ASD pathophysiology, with time, the data may converge on a unified mechanistic model for ASD, facilitating development of therapeutic interventions (Casci, 2011).

## Future directions

Over the last decade great progress has been made in establishing methods for generation of cortical projection neurons or inhibitory interneurons from hPSCs, but many challenges remain. Methods for the generation of layer- or subregion-specific cortical neurons from hPSCs would be beneficial for studies of ASD pathophysiology. Impairment of specific cortico-striatal (CStr) connectivity has been implicated in ASD, and many ASD-associated genes are involved in CStr synapses (reviewed by Shepherd, 2013). A recent study also showed that differences in gene expression between the frontal and temporal cortices in the normal brain are significantly attenuated in the autistic brain, which implies altered cortical patterning (Voineagu et al., 2011). This finding supports the notion that layer- or subregion-specific neuronal subtypes would be tremendously valuable for *in vitro* modeling of ASD. Although a direct method for layer- or sub-regional specific cortical neurons from hPSCs has not been developed yet, accumulating evidence from studies on mESC differentiation and mouse development suggest possible approaches for achieving this goal (Eiraku et al., 2008).

Differentiation of functionally mature neurons from hPSCs is a long process with multiple steps requiring a few months. This may increase heterogeneity of the final neuronal population, even if the protocol was intended to enrich for a specific neuronal subtype. One way to overcome these difficulties is to convert patient-derived somatic cells directly into neurons, skipping cellular reprogramming and differentiation. A recently introduced method for direct conversion of fibroblasts to functional cortical neurons relies on forced expression of neural-lineage specific transcription factors (Vierbuchen et al., 2010; Pang et al., 2011). The low conversion efficiency (2–4% of cells) of the method is a major obstacle for disease modeling, although small molecule-based modulation reportedly improved differentiation efficiencies to ~80% (Ladewig et al., 2012). Regardless of differentiation efficiencies, the disease modeling potential for direct conversion from fibroblasts to terminally differentiated neurons is limited by the number of patient-derived somatic cells that are available. Südhof and colleagues recently developed a robust and simple method for conversion of hPSCs to functional cortical neurons with 100% efficiency in 3 weeks by expressing a single transcription factor (Zhang et al., 2013). This approach is not limited by available cell numbers, but it does require pre-existing patient-specific iPSC lines for disease modeling. Despite the method's robustness and feasibility, one should be cautious in utilizing direct conversion for disease modeling for ASD because it skips the normal developmental process, which may be critical for manifestation of ASD-associated phenotypes (Sandoe and Eggan, 2013). Furthermore, forced expression of key transcription factors may override pathological mechanisms underlying ASD (Brennand and Gage, 2012). Therefore, it is more desirable to use direct conversion approaches as a complement for screening disease phenotypes or to reinforce results obtained by neurons differentiated from hiPSCs.

Significant line-to-line variability has been observed in the neuronal differentiation of hPSCs (Wu et al., 2007; Hu et al., 2010; Kim et al., 2010) and efforts have been made to overcome this issue. One suggestion for bypassing such variation among iPSC lines is to pre-screen iPSC lines to select those with good responsiveness to lineage specification procedures (Bock et al., 2011; Boulting et al., 2011). However, reduced neural differentiation/specification may be a biologically relevant phenotype in studies of ASD, which would be unintentionally excluded by using this screening approach (Sandoe and Eggan, 2013). Melton et al. recently showed that priming human iPSCs with 1–2% demethylsulfoxide (DMSO) prompted exit from the cell cycle and improved differentiation efficiency of hiPSCs (Chetty et al., 2013). Given the recent evidence that the cell cycle is highly implicated in maintenance of pluripotency and fate decision, and elaborate modulation of cell cycle leads to lineage specification from hPSCs, this strategy may provide a solution for taming the variation in differentiation resulting from cell line-specific characteristics (Pauklin and Vallier, 2013). However, it is important to determine whether specific ASD-associated genetic variations influence cell cycle progression prior to applying these methods.

Finally, neuronal differentiation *in vitro* may not fully recapitulate neuronal development as it happens *in vivo*. Proliferation and differentiation of cortical progenitors occurs within specific niche environments characterized by signaling from the VZ, differentiated daughter cells, as well as signaling from non-neural sources, such as astrocytes, blood vessels, meninges (reviewed by Johansson et al., 2010), and microglia (Antony et al., 2011). The contribution of vascular endothelial cells to cortical development has been appreciated for a decade (Shen et al., 2004). Furthermore, increasing evidence suggests that microglial dysregulation may underlie several neuropsychiatric conditions including ASD (reviewed by Frick et al., 2013). Therefore, the absence of non-neuronal components during *in vitro* differentiation culture may obscure disease-relevant phenotypes using neurons generated from hPSCs. In line with this idea, it would be quite informative to determine whether co-culturing healthy cortical neurons with endothelial cells or microglia derived from individuals with ASD impairs neuronal function. Astrocytes are often supplied during the maturation of hPSC-derived neurons, as they promote synaptogenesis (Johnson et al., 2007). However, astrocytes contribute to the pathophysiology of neurodevelopmental disorders. Indeed, hiPSC-derived RTT astrocytes adversely affect the function of control neurons (Williams et al., 2014). The availability of protocols for generating hiPSC-derived astrocytes will allow co-culture experiments to examine the role of astrocytes in neuronal dysfunction associated with ASD. Finally, investigation of niche effects may help determine optimal *in vitro* conditions for cortical differentiation, and provide clues for therapeutic approaches.

## Conclusion

With rapid progress in our ability to precisely manipulate hiPSCs, the tremendous knowledge gap between ASD genetics and our understanding of its pathophysiology is beginning to close. Using iPSC technology, it is possible to generate limitless supplies of human ASD-specific cortical neurons, which can revolutionize experimental analyses of ASD. Already, studies of the neurodevelopmental disorders RTT, TS, AS, and PMDS have shown that neuronal phenotypes can be identified using iPSC-derived neurons, and that these phenotypes can be corrected. Given the genetic heterogeneity of idiopathic ASD and the diversity in its clinical presentation, robust and highly reproducible methods for hiPSC manipulation is essential for linking genotype to phenotype. With the advent of facile mammalian genome engineering methods (reviewed in Hsu and Zhang, 2012; Mali et al., 2013) allowing for generation of gene-corrected cells from patient hiPSCs, precise neuronal differentiation methods will greatly facilitate the determination of causal mechanisms underlying ASD pathophysiology. However, there is still a great need for optimized and standardized cortical differentiation protocols that are efficient, swift, scalable, and produce desired neuronal subpopulations. Upon identification of ASD-associated neuronal phenotypes, iPSC-derived cortical neurons may be used for screens of chemical libraries, which will greatly facilitate drug discovery. With continued progress in neuronal differentiation from hiPSCs, the stage is set for understanding how ASD develops and how it may be treated.

### Conflict of interest statement

The authors declare that the research was conducted in the absence of any commercial or financial relationships that could be construed as a potential conflict of interest.
